# Antibacterial Peptide CecropinB2 Production via Various Host and Construct Systems

**DOI:** 10.3390/molecules21010103

**Published:** 2016-01-16

**Authors:** Wei-Shiang Lai, Shu-Chen Kan, Chia-Chi Lin, Chwen-Jen Shieh, Yung-Chuan Liu

**Affiliations:** 1 Department of Chemical Engineering, National Chung Hsing University, 250 Kuo Kuang Road, Taichung 40227, Taiwan; s099065033@gmail.com (W.-S.L.); sherrykan0402@gmail.com (S.-C.K.); teddy@ms23.url.com.tw (C.-C.L.); 2 Biotechnology Center, National Chung Hsing University, 250 Kuo Kuang Road, Taichung 40227, Taiwan; cjshieh@nchu.edu.tw

**Keywords:** cecropinB2, antibacterial peptides, multi-drug-resistant, *Pichia pastoris*, *Bacillus subtilis*

## Abstract

Cecropin is a cationic antibacterial peptide composed of 35–39 residues. This peptide has been identified as possessing strong antibacterial activity and low toxicity against eukaryotic cells, and it has been claimed that some types of the cecropin family of peptides are capable of killing cancer cells. In this study, the host effect of cloning antibacterial peptide cecropinB2 was investigated. Three different host expression systems were chosen, *i.e.*, *Escherichia coli*, *Bacillus subtilis* and *Pichia pastoris*. Two gene constructs, cecropinB2 (cecB2) and intein-cecropinB2 (INT-cecB2), were applied. Signal peptide and propeptide from *Armigeres subalbatus* were also attached to the gene construct. The results showed that the best host for cloning cecropinB2 was *P. pastoris* SMD1168 harboring the gene of pGAPzαC-prepro-cecB2 via Western blot confirmation. The cecropinB2 that was purified using immobilized-metal affinity chromatography resin showed strong antibacterial activity against the Gram-negative strains, including the multi-drug-resistant bacteria *Acinetobacter baumannii*.

## 1. Introduction

In 1929, Alexander Fleming identified penicillin, the first chemical compound with antibiotic properties [1]. More and more antibiotic compounds were then found and used in the medical field. Due to the antibiotics’ overuse, multi-drug-resistant bacteria have become a big issue for treatment with antibiotics [2]. Using antibacterial peptides was believed to be a good way to eliminate the multi-drug resistant (MDR) bacteria issue in the medical field [3,4,5].

Cecropins are a family of cationic antibacterial peptides and have six isoforms (A–F) with the common homologous sequences of 35–39 amino acids [6]. These cecropins, originally found in the immune hemolymph of skin worm pupae, have a broad antibacterial spectrum against the Gram-negative and Gram-positive bacteria, and even against the MDR bacteria [3,7]. Experimental results also showed that they possessed an anti-tumor effect without damaging the eukaryotic cells [3,4,8]. Therefore, they might have high potential for the application in medicine, agriculture and animal husbandry.

To date, various constructs have been established for production of antibacterial peptides. However, difficulties have been encountered in peptide expression because the toxicity of antibacterial peptide might be toxic to its expression hosts [9,10]. The host strains play an important role in the process of efficiently expressing large amounts of recombinant antibacterial peptides. The most commonly-used host strains, *E. coli*, *B. subtilis* and *P. pastoris*, have their individual advantages and disadvantages [11,12,13]. To conquer the difficulty of host self-destruction by the toxic target peptides, the fusion protein method was proposed to reduce the toxicity. This method has been used for many peptides expression, including thioredoxin [10], green fluorescent protein (GFP) [14], small ubiquitin-related modifier (SUMO) [15], and cationic elastin-like polypeptides (CELP) [16]. In this study, intein (INT) was used as the fusion peptide to reduce the toxicity of the antibacterial cecropin B2.

In this study, three hosts, *E. coli*, *B. subtilis* and *P. pastoris*, were chosen, and two target peptide genes, cecB2 and INT-cecB2, were carried out to compare their effect on cecropinB2 expression. *Armigeres subaltatus* mosquitoes are the original source of the peptide cecropinB2. In order to help the transport and folding of the target cecropinB2, signal peptide and propeptide from *Armigeres subalbatus* were also applied to the gene constructs with host *P. pastoris* SMD1168. The best production construct was selected and the antibacterial activity was tested for the obtained purified cecropinB2. This study revealed the host effect on antibacterial peptide cecropinB2 production.

## 2. Results and Discussion

### 2.1. CecropinB2 Production in E. coli

**Figure 1:**
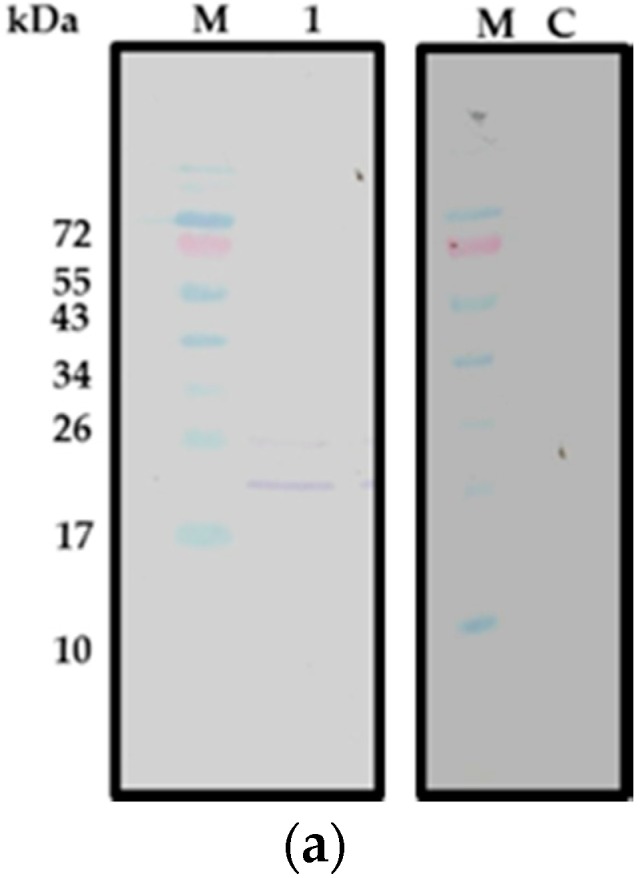

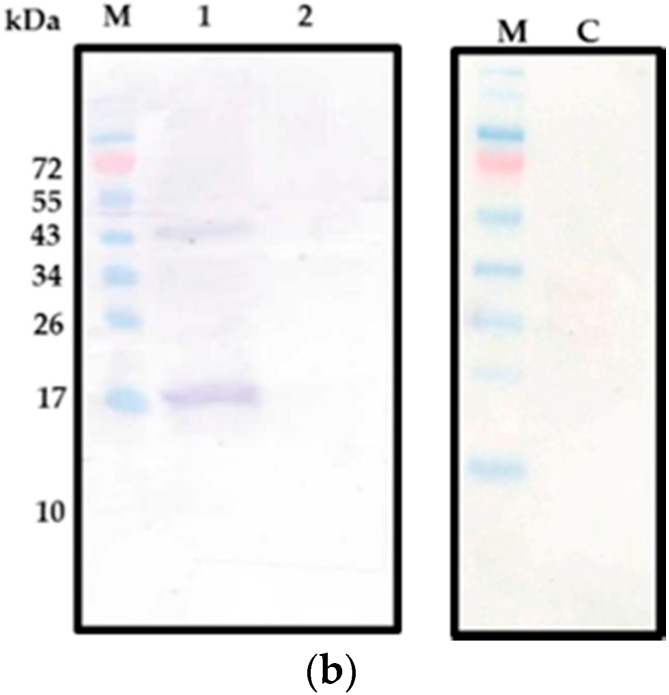
Expression of cecropinB2 in *E. coli* ER2566 and *B. subtilis* WB800. (**a**) Western blot of pET26b-INT-cecB2/*E. coli* ER2566; where Lane M, molecular weight markers (kDa); Lane 1, supernatant; Lane C: control (supernatant of ET26b/*E. coli* ER2566); The cell debris was centrifuged at 7000 *g* at 4 °C for 10 min to obtain the supernatant (Lane 1); (**b**) Western blot of PRPA-cecB2/*B. subtilis* WB800; where Lane M, molecular weight markers (kDa); Lane 1, cell crude extract; Lane 2, 100× concentrated extracellular fluid; Lane C: control (cell crude extract of PRPA-/*B. subtilis* WB800). The culture broth was centrifuged at 6000× *g* at 4 °C for 10 min to obtain the cell and extracellular fluid.

To investigate the expression of cecropinB2 in *E. coli*, five constructs (pET26b-cecB2, pET28a-cecB2, pET28a-INT-cecB2, pET26b-INT-cecB2, pET26b-10K-INT-cecB2 and pET26b-10R-INT-cecB2, where 10K = 10 lysines, 10R = 10 arginines) were transformed into *E. coli* ER2566. The individual strain was cultivated and induced with 1 mM IPTG at 25 °C for 6 h. The results showed that three constructs, pET26b-cecB2, pET28a-cecB2 and pET-28a-INT-cecB2, did not express the target peptide, whereas the construct of pET26b-INT-cecB2/*E. coli* ER2566 did successfully express the target peptide as confirmed by Western blot (Figure 1a). The upper band of Figure 1a, lane 1 with the molecular weight of 23.5 kDa might be the incomplete cleavage segment of the signal peptide pelB (MW: 2.5 kDa) of the pET26b plus the cecropinB2 and INT (about 21 kDa). The results indicated that the target protein of INT-cecB2 might possess lower toxicity than cecropinB2. In addition, the plasmids affected the production of antibacterial peptides according to the result of pET26b (which can be produced with the construct pET26b-INT-cecB2) and pET28a (no production with the construct pET28a-INT-cecB2) systems, suggesting that the target peptide might give lower toxcity to the host when the peptide can be transferred to periplasm (pET26b). Moreover, to increase the solubility of the target protein, the 10K and 10R segments [17] were added to the N-terminal of INT-cecB2 and the results were observed by Western blot (data no shown). The addition of 10K and 10R segments did not improve the solubility of INT-cecB2, suggesting that the toxicity of target peptides (INT-cecB2) still had some effect on limiting the protein solubility. The antibacterial peptide cecropinB2 did not express well in *E. coli* ER2566. This might be due to the peptide’s toxicity against its host. The fusion protein approach by adding an INT seemed to slightly improve the expression; however, the production of INT-cecB2 was at a low level (<10 mg/L).

### 2.2. CecropinB2 Production in B. subtilis

To study the expression of cecB2 in *B. subtilis*, two constructs, PRPA-cecB2 and PRPA-INT-cecB2, were transformed into the *B. subtilis* WB800. The expression results were confirmed by Western blot. The construct of PRPA-INT-cecB2 in *B. subtilis* WB800 did not produce any target protein (INT-cecB2), indicating that the host *B. subtilis* could not express the INT protein well. The construct of PRPA-cecB2 in *B. subtilis* WB800 was found capable of expressing the target peptide; however, the expression was intracellular with very low yield (<10 mg/L), suggesting the highly-positive-charge property of the target peptide might still affect host’s secretion and expression. The larger molecular weight (17 kDa) shown in Western blot (Figure 1b) was likely the molecular size of the signal peptide (13 kDa) plus cecropinB2 (4 kDa). However, because of the low content of target protein, a longer staining time was needed, which resulted in the unspecific band observed in 43 kDa.

### 2.3. CecropinB2 Production in P. pastoris

**Figure 2:**
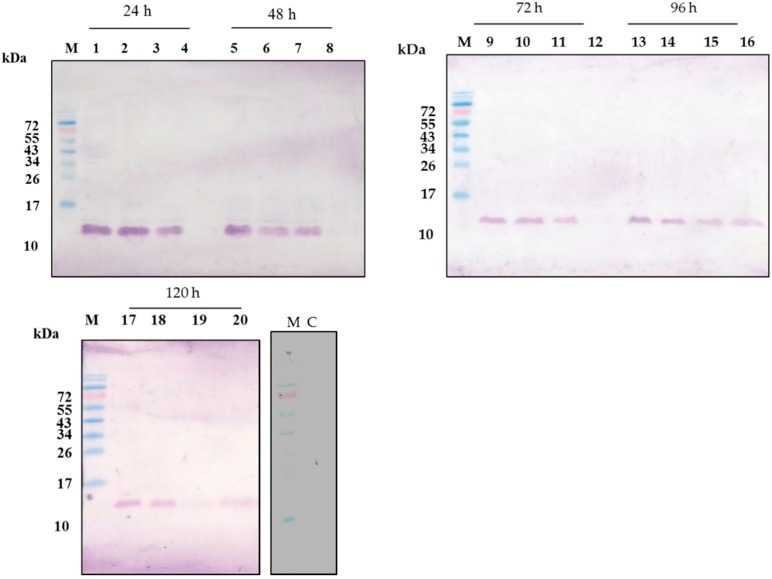
Western blot of pGAPZaC-prepro-cecB2/*P. pastoris* SMD1168. Lane M, molecular weight markers (kDa); Lane C: control (cell crude extract of pGAPZaC/*P. pastoris* SMD1168) lane 1–4, crude extract, pellet, supernatant, 100× concentrated extracellular fluid, at 24; lane 5–8, the same items at 48 h; lane 9–12, at 72 h; lane 13–16, at 96 h; lane 16–20, at 120 h cultivation time.

To study the cecropinB2 expression in *P. pastoris*, three constructs, pGAPZαC-cecB2, pGAPZαC-INT-cecB2 and pGAPZαC-prepro-cecB2, were transformed into the host *P. pastoris* SMD1168. The construct of pGAPZαC-INT-cecB2 in host *P. pastoris* SMD1168 could not translate the target protein (INT-cecB2) well (data not shown). The protein INT originally discovered from bacteria might prohibit the expression in *P. pastoris*. In contrast, the constructs of pGAPZαC-cecB2 and pGAPZαC-prepro-cecB2 in host *P. pastoris* SMD1168 could properly express the target peptide. Among them, the construct pGAPZαC-prepro-cecB2 could express the target peptide with its signal peptide intracellularly to a high soluble level within 24 h (>80 mg/L) (Figure 2a). Meanwhile, the target peptides could be secreted out to the culture broth for a 96 h cultivation (Figure 2b). The target peptides with their highly positive charge might exhibit a large affinity to the host’s inner membrane (nagative-charge), which might cause the delay of the cecropinB2 secretion as shown in the time course of the production. For a longer cultivation time of 120 h, both the intracellular and extracellular target protein contents were significantly decreased, suggesting that the cells might gradually decompose the target protein (Figure 2c).

### 2.4. Comparison of the Host Systems

The cecropinB2 production by applying various host strains of *E. coli*, *B. subtilis* and *P. pastoris* gave different results in the expression. The fusion protein approach could reduce the host directed toxicity of cecropinB2 and was successfully expressed in host *E. coli*. In contrast, it had a negative effect on hosts *B. subtilis* and *P. pastoris*. Comparison of cecropinB2 production by three expression systems showed that the highest yield occurred when using *P. pastoris* SMD1168 harboring the pGAPZαC-prepro-cecB2 (Table 1c). The highest yield was about 90 mg/L for a cultivation time of 24 h. This was better than that of the same construct pGAPZαC-cecB2 without prepro peptide (41 mg/L). Other constructs in this study gave yields lower than 40 mg/L. Based on the aforementioned results, it might be concluded that the secretion systems for *B. subtilis* and *P. pastoris* were greatly affected by the highly-positive-charge antibacterial peptide cecropinB2. The affinity between cecropinB2 and the cell membrane might build a barrier to limit the secretion process. This study also illustrated that, by properly constructing the host and plasmid system, the antibacterial peptide cecropinB2 production could be achieved.

**Table 1:** Yield of cecropinB2 under various expression systems.

Construct	Total Protein Content (mg/L) ^1^	Purity (%) ^2^	Yield of CecropinB2 ^3^
(a) *E. coli*			
pET26b-cecB2/*E. coli* ER2566	-	-	-
pET-28a-cecB2/*E. coli* ER2566	-	-	-
pET28a-INT-cecB2/*E. coli* ER2566	-	-	-
pET26b-INT-cecB2/*E. coli* ER2566	1022	0.8	+
pET26b-10K-INT-cecB2/*E. coli* ER2566	987	0.8	+
pET26b-10R-INT-cecB2/*E. coli* ER2566	1055	0.8	+
(b) *B. subtilis*			
PRPA-cecB2/*B. subtilis* WB800	1128	1.8	+
PRPA-INT-cecB2/*B. subtilis* WB800	-	-	-
(c) *P. pastoris*			
pGAPZαC-cecB2/*P. pastoris* SMD1168	1980	2.4	++
pGAPZαC-INT-cecB/*P. pastoris* SMD1168	-	-	-
pGAPZαC-prepro-cecB2/*P. pastoris* SMD1168	2048	4.3	+++

^1^ Total protein content was determined by the Bradford protein assay; symbol -: no detection; ^2^ Purity was determined by the image scanning; symbol -: no detection; ^3^ Definition of yield: purity of target protein (qualified via image scanning) × total protein content (quantified via Bradford); symbols -: no production; +: yield > 1 mg/L; ++: yield > 40 mg/L; +++: yield > 80 mg/L.

### 2.5. Antibacterial Tests

**Figure 3:**
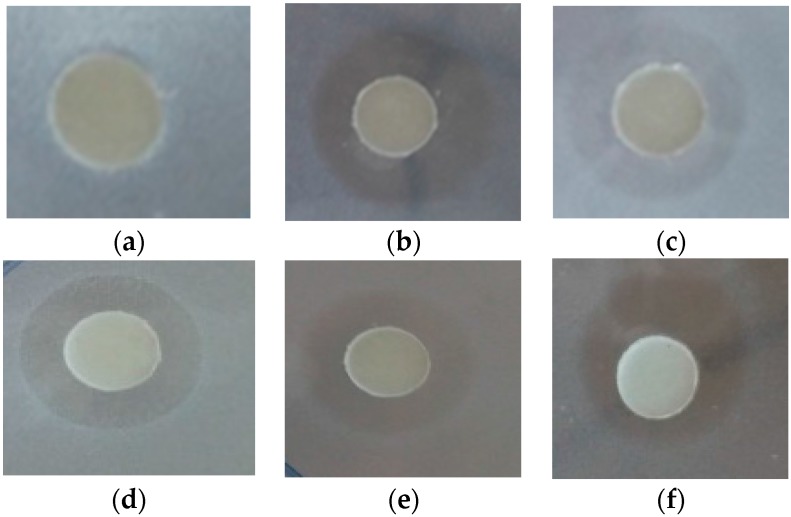
Antibacterial activity with agar diffusion test; where (**a**) elution buffer as blank; (**b**–**f**) inhibition zone against *E. coli* ER2566, BL21, Rosetta, JM109 and DH1, respectively. The testing solution contains purified cecropinB2 (10 μg/mL) in the elution buffer.

To check the antimicrobial activity effect, the purified cecropinB2 and the synthetic cecropinB2 were used to test the antibacterial activity, respectively. In the primary tests, the whole construct without gene CecB2 was expressed in the same host which didn’t show any activity against sensitive bacterial cells, indicating the fragments or chimeras did not respond to the antibacterial activity. In addition, the elution buffer and the extraction of host *P. pastoris* also displayed none of the inhibitory effect (data not shown). All these results gave evidence to the correct expression of cecropinB2 in the purified product. Figure 3 shows the antibactrial test results for *E. coli* strains. Various strains, *i.e.*, *E. coli* strains (in Table 2), *B. subtilis* WB800, *P. pastoris* SMD1168, *A. baumannii* BCRC 15884 and *A. baumannii* E1359 (MDR) were used (Table 2). The results showed the purified cecropinB2 possessing the same activity as the synthetic peptides, which demonstrated strong antibacterial activity against the Gram-negative strains even in the case of *A. baumannii*; however, it did not exhibit toxicity toward the Gram-positive strains and eukaryotic cells such as *P. pastoris* (Table 2).

**Table 2:** Antibacterial spectrum of purified cecropinB2 ^1^.

Bacteria Strain	Antibacterial Activity ^2^	
	Purified CecropinB2 (10 μg/mL)	Synthetic CecropinB2 (4 μg/mL)
*E. coli* ER2566	6.5	6.8
*E. coli* BL21	6.8	7.2
*E. coli* Rosetta	7.1	7.2
*E. coli* JM109	6.5	6.6
*E. coli* DH1	6.5	6.7
*B. subtilis* WB800	-	-
*P. pastoris* SMD1168	-	-
*A. baumannii* BCRC 15884	7.2	6.8
*A. baumannii* E1359	7.3	6.9

^1^ The cecropinB2 was purified from the host *P. pastoris* SMD1168 with the construct of pGAPZαC-prepro-cecB2; ^2^ Antibacterial activity was determined by the diameter (mm) of inhibition zone; -: no clear zone observed. Concentration of the purified cecropinB2 was determined via the Bradford assay and image scanning result.

## 3. Materials and Methods

### 3.1. Bacterial Strains, Plasmids and Synthetic CecropinB2

Four strains, *i.e.*, *E. coli* strains DH5α, ER2566, *B. subtilis* strain WB800 and *P. pastoris* strain SMD1168, were used as the hosts for recombinant DNA manipulation and recombinant protein expression. The gene templates of antibacterial peptide pre-pro-cecropinB2 and plasmid PRPA were obtained, respectively, from Prof. Kuang-Hui Lu and Prof. Yeh Chuan-Mei [18] at the National Chung Hsing University, Taiwan. *B. subtilis* WB800 and *P. pastoris* SMD1168 were obtained from Prof. Chieh-Chen Huang and Prof. Huang Chien-jin at National Chung Hsing University, Taiwan, respectively. The synthetic cecropinB2 was purchased from Neogene Biomedicals Corp (Taiwan) as the standard. The number of amino acid and theoretical molecular mass of protein or peptide used in this study were summarized and are shown in Table 3.

**Table 3:** Number of amino acid and theoretical molecular mass.

Protein	Number of Amino Acid	Theoretical Molecular Mass (Da)
CecropinB2	36	3749
INT-cecB2	193	17,569
signal peptide (SP)	18	2085
Propeptide	5	468

### 3.2. Construction of Expression Systems

**Figure 4:**
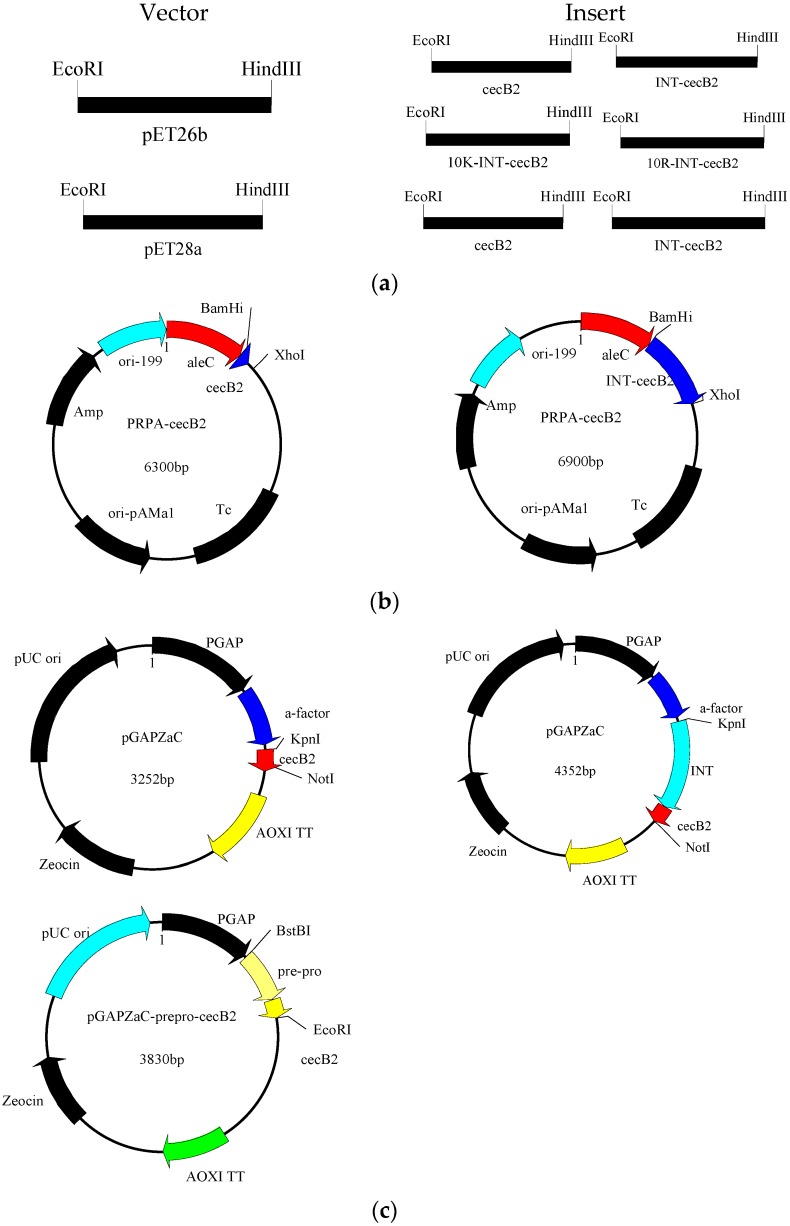
Plasmid constructs in this study. (**a**) Plasmid constructs in *E. coli*; (**b**) Plasmid constructs in *B. subtilis*; (**c**) Plasmid constructs in *P. pastoris*.

The templates of prepero-cecropinB2 and plasmid pTWIN (New England Biolabs, Ipswich, MA, USA) were used to conduct an overlap extension PCR to obtain cecB2, pre-pro-cecropinB2 and INT-cecB2 which were further ligated to the vector pET26b, pET28a, PRPA and pGAPZaC, respectively. To increase the solubility, the amino acid segments of 10K and 10R were also used to fuse in the N-terminal of INT-cecB2 and ligated to the vector pET26b. The major plasmid constructs used in the study are showed in Figure 4. All recombinant DNA manipulations were performed following the standard procedures [19].

### 3.3. Production of the Antibacterial Peptide

#### 3.3.1. Culture of *E. coli*

The plasmid-transformed *E. coli* was grown overnight in 5 mL of Luria-Bertani (LB) medium, which was used to inoculate the main culture containing 100 mL of LB medium with 50 μg/mL kanamycin. The main culture was grown at 37 °C until OD600 reached 0.6. The cultivation was induced with 1 mM isopropyl-β-d-thiogalactopyranoside (IPTG) at 25 °C for another 6 h. Cells were then collected by centrifugation (6000× *g*, 5 min) and stored at 4 °C for further analysis.

#### 3.3.2. Culture of *B. subtilis*

The plasmid-transformed *B. subtilis* was grown overnight in 5 mL of LB medium. The harvested broth was used to inoculate the main culture containing 100 mL of LB medium containing 10 μg/mL Tetracycline. The culture was grown at 30 °C for 24 h. Cells were then collected by centrifugation (6000× *g*, 5 min) and stored at 4 °C for further analysis.

#### 3.3.3. Culture of *P. pastoris*

The plasmid-transformed *P. pastoris* was grown overnight in 5 mL of LB medium as the seed to inoculate 100 mL of YPD medium containing 50 μg/mL zeocin. The culture was grown at 30 °C for 120 h. The culture at different time intervals was collected and separated into cells and medium by centrifugation (6000× *g*, 5 min) and stored at 4 °C for further analysis.

### 3.4. Purification of Target Protein

The harvested cell pellet, with the the construct of pGAPZαC-prepro-cecB2/*P. pastoris* SMD1168, was re-suspended in Tris-HCl buffer (20 mM tris-HCl, pH 8.0, 50 mM NaCl) and then lysed by sonication using an ultrasonic processor at 20 W for 10 cycles (30 s working, 30 s free) to obtain the cell crude extract. The cell debris was pelleted at 6500 *g* at 4 °C for 20 min to obtain the supernatant and pellet. The supernatant was collected and applied to an immobilized-metal affinity chromatography resin (Ni^2+^-chelating) column. After being washed with the washing buffer (20 mM tris-HCl, pH 8.0, 50 mM NaCl, 20 mM imidazole), the product cecropinB2 was eluted with the elution buffer (20 mM tris-HCl, pH 8.0, 50 mM NaCl, 200 mM imidazole) and stored at 4 °C for futher use.

### 3.5. Analytical Methods

Protein analysis was carried out using 15% sodium dodecyl sulfate-polyacrylamide gel electrophoresis (SDS-PAGE) and coomassie blue staining according to the method of Laemmli [20]. For Western blot, the protein samples run on SDS-PAGE were transferred onto PVDF membrane. After being blocked with 1% BSA/TTBS (20 mM tris-HCl, pH 7.5, 0.9% NaCl, 0.1% tween-20) buffer, the membrane was washed three times with TTBS buffer and incubated with anti-His tag primary antibody (1:10,000) in 0.5% BSA/TTBS buffer at room temperature overnight. Then, the membrane was washed with TTBS buffer three times and incubated with anti-goat tag secondary antibody (1:10,000) in 0.5% BSA/TTBS buffer at room temperature for 2 h. After the three washings, staining was performed using the BCIP/NBT chromogenic system. The Bradford protein assay was used for total protein content measurement [21] and imageJ software (version 1.49s, NIH, Bethesda, MD, USA) was used for purity determination [22]. The yield of cecropinB2 was determined by multiple of the protein content with its purity.

### 3.6. Antibacterial Assay

Antibacterial activity was analyzed using the agar diffusion test [23]. Some 100 mL of LB broth containing 0.8% agar (LB agar) was autoclaved and then cooled to a temperature of 45 °C, followed by instantly adding an aliquot of 166 μL tested bacteria dilution solution (OD_600_ = 0.5, where OD_600_ represents the cell weight concentration). The 6 mL LB agar was poured over a 9.0 cm petri dish, giving an agar depth of 1 mm. After the agar solidified, 20 μL of the purified cecropinB2 was added to a paper disc (6 mm in diameter.) on the surface of the agar. The disc was incubated at 37 °C for 12 h. Antibacterial activity was indicated by the clear zone around the testing point. The antibacterial activity against MDR *A. baumannii* was tested in the experimental lab of Prof. Wu-Chun Tu at National Chung Hsing University, Taiwan.

## 4. Conclusions

In this study, three host strains were used to produce the antibacterial peptides. It was found that the relative yield of the cecropin B2 production could be rescribed in the order: *P. pastoris* > *B. subtillis* > *E. coli*. The best construct pGAPZαC-prepro-cecB2/*P. pastoris* SMD1168 could produce the antibacterial peptides within 24 h intracellularly, and the purified cecropinB2 showed strong antibacterial activity against the Gram-negative strains and even against that of MDR *A. baumannii*.
